# High Speed, High Density Intraoperative 3D Optical Topographical Imaging with Efficient Registration to MRI and CT for Craniospinal Surgical Navigation

**DOI:** 10.1038/s41598-018-32424-z

**Published:** 2018-10-05

**Authors:** Raphael Jakubovic, Daipayan Guha, Shaurya Gupta, Michael Lu, Jamil Jivraj, Beau A. Standish, Michael K. Leung, Adrian Mariampillai, Kenneth Lee, Peter Siegler, Patryk Skowron, Hamza Farooq, Nhu Nguyen, Joseph Alarcon, Ryan Deorajh, Joel Ramjist, Michael Ford, Peter Howard, Nicolas Phan, Leo da Costa, Chris Heyn, Gamaliel Tan, Rajeesh George, David W. Cadotte, Todd Mainprize, Albert Yee, Victor X. D. Yang

**Affiliations:** 1 Department of Biomedical Physics, Ryerson University, Toronto, ON Canada; 2 Biophotonics and Bioengineering Laboratory, Ryerson University Sunnybrook Health Sciences Centre, Toronto, ON Canada; 3 Division of Neurosurgery, Department of Surgery, University of Toronto, Toronto, ON Canada; 4 Institute of Medical Science, School of Graduate Studies, University of Toronto, Toronto, ON Canada; 5 Department of Electrical and Computer Engineering, Ryerson University, Toronto, ON Canada; 6 Division of Orthopedic Surgery, Department of Surgery, University of Toronto, Toronto, ON Canada; 7 Division of Neuroradiology, Department of Medical Imaging, Sunnybrook Health Sciences Centre, Toronto, ON Canada; 8 Department of Medical Imaging, University of Toronto, Toronto, ON Canada; 9 Jurong Health, Ng Teng Fong General Hospital, Singapore, Singapore; 10 Spine Program and Division of Neurosurgery, Department of Clinical Neurosciences, Department of Radiology, University of Calgary, Calgary, Canada; 11 Hotchkiss Brain Institute, Cumming School of Medicine, University of Calgary, Calgary, Canada

## Abstract

Intraoperative image-guided surgical navigation for craniospinal procedures has significantly improved accuracy by providing an avenue for the surgeon to visualize underlying internal structures corresponding to the exposed surface anatomy. Despite the obvious benefits of surgical navigation, surgeon adoption remains relatively low due to long setup and registration times, steep learning curves, and workflow disruptions. We introduce an experimental navigation system utilizing optical topographical imaging (OTI) to acquire the 3D surface anatomy of the surgical cavity, enabling visualization of internal structures relative to exposed surface anatomy from registered preoperative images. Our OTI approach includes near instantaneous and accurate optical measurement of >250,000 surface points, computed at >52,000 points-per-second for considerably faster patient registration than commercially available benchmark systems without compromising spatial accuracy. Our experience of 171 human craniospinal surgical procedures, demonstrated significant workflow improvement (41 s vs. 258 s and 794 s, p < 0.05) relative to benchmark navigation systems without compromising surgical accuracy. Our advancements provide the cornerstone for widespread adoption of image guidance technologies for faster and safer surgeries without intraoperative CT or MRI scans. This work represents a major workflow improvement for navigated craniospinal procedures with possible extension to other image-guided applications.

## Introduction

**Figure 1:**
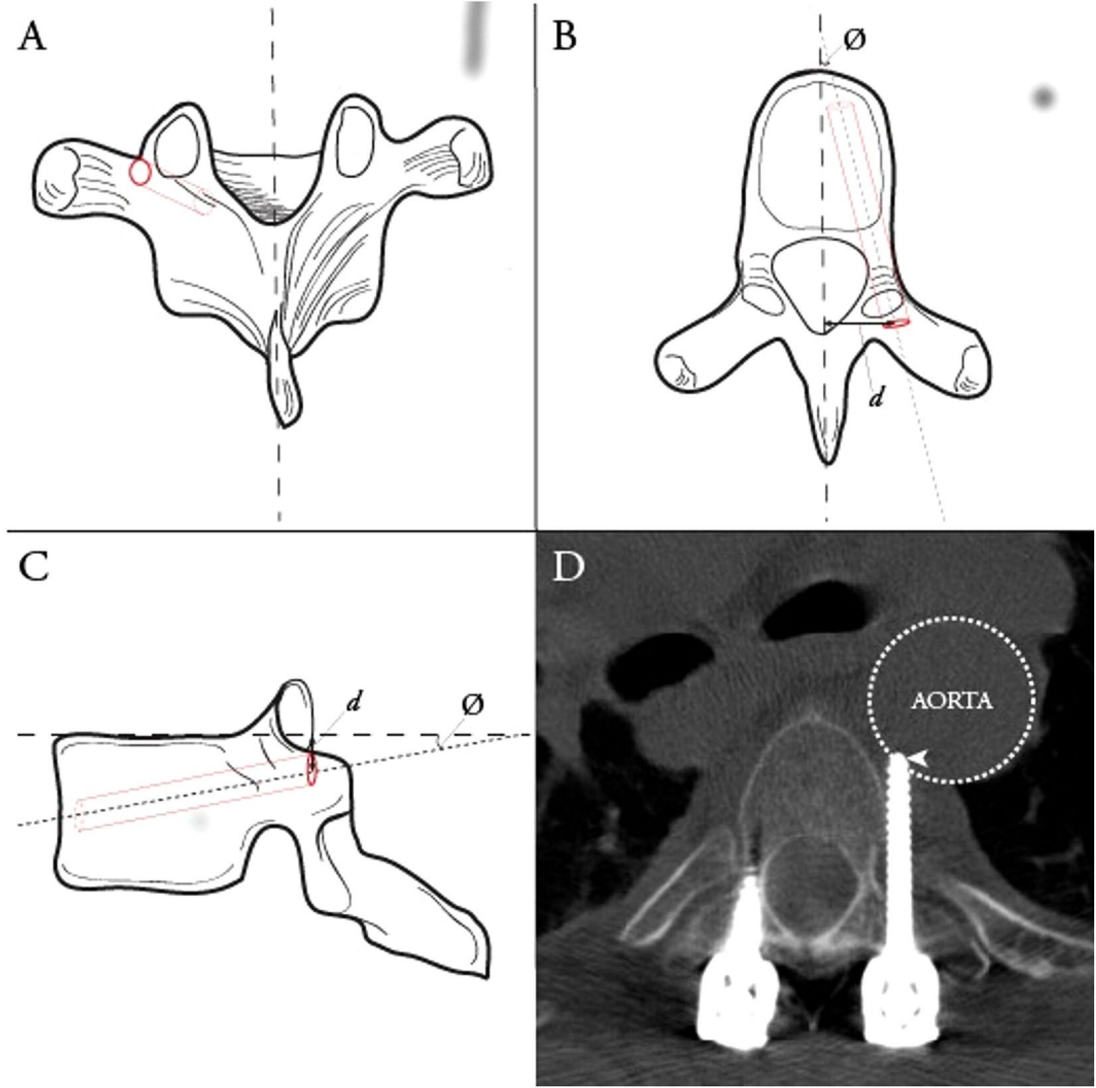
Ideal thoracic pedicle screw entry point and trajectory. Ideal thoracic pedicle screw entry point (dark red circle) and trajectory (dashed red cylinder) in the coronal (**A**), axial (**B**) and sagittal (**C**) planes. Ideal entry point distance (d) and trajectory angle (∅) shown on axial and sagittal planes. Example of a misplaced thoracic pedicle screw via freehand technique (**D**), Heary Grade V, with tip (arrowhead) abutting the aorta.

**Figure 2:**
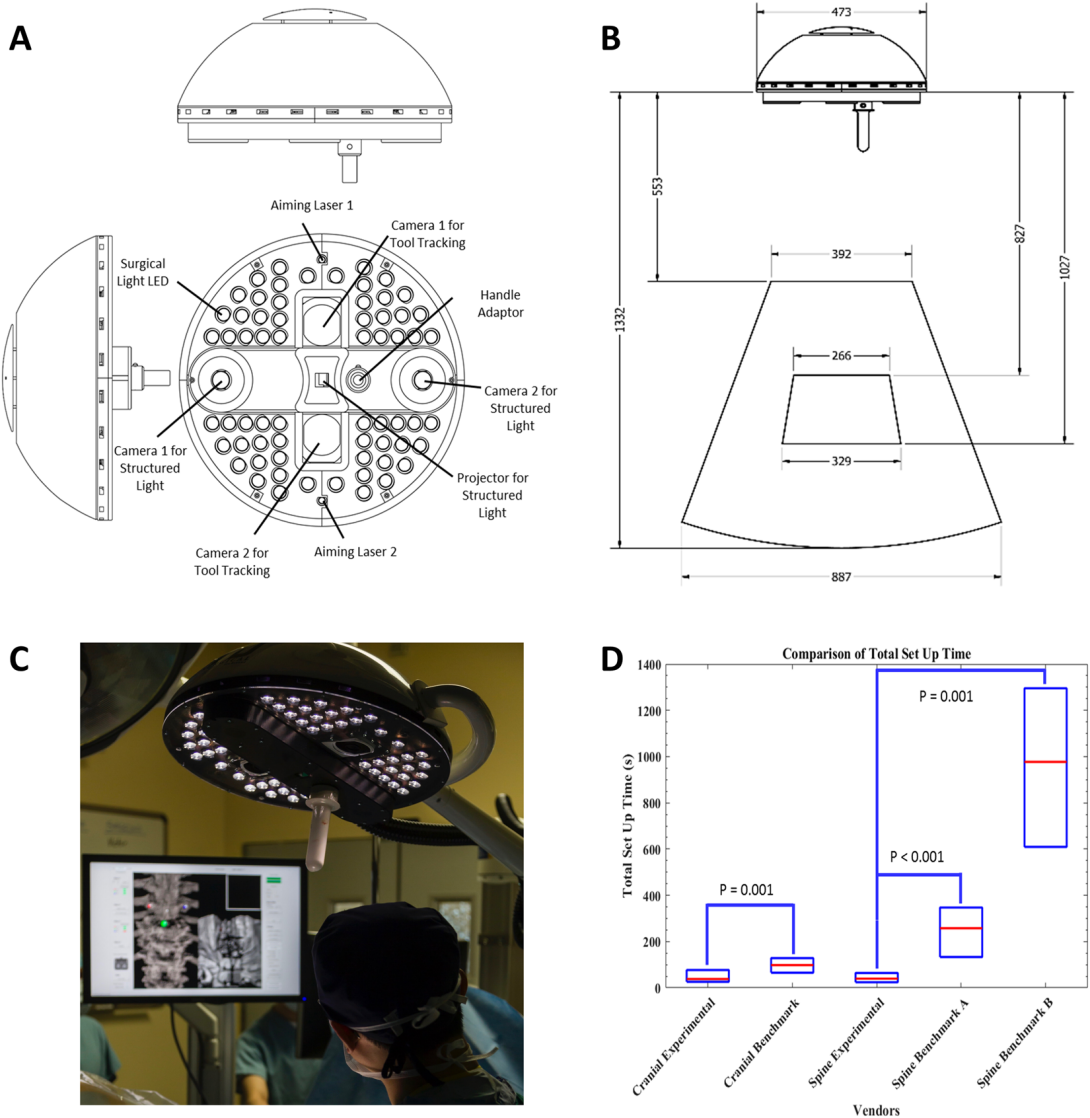
Clinical prototype of the experimental navigational system. (**A**) Design model of the surgical light head with embedded navigation. Designed to inconspicuously serve as traditional boom-supported surgical light head comprised of 64 high intensity surgical light LEDs to provide standard lighting with minimal spectral overlap with the navigation optics. Binocular infrared cameras utilizing provide real-time tracking of passive-reflective markers mounted on surgical tools. A digital mirror device centered around binocular structured light cameras forming an epipolar baseline provide intra-operative surface imaging for registration to the pre-operative images. Co-ordinates of the tracked tools are easily matched to the acquired structured light surface image. (**B**) Design model of the surgical light head with embedded navigation: Technical specifications: Field of view of the infrared tracking volume (outer pyramid) and the structured light imaging volume (inner pyramid). All measurements are in millimeters. (**C**) Prototype navigation system in clinical use. (**D**) Comparison of total setup time (median and IQR) for cranial and spine applications of experimental and benchmark navigation systems (cranial: StealthStation; spine A: Nav3/3i; spine B: O-arm).

**Figure 3:**
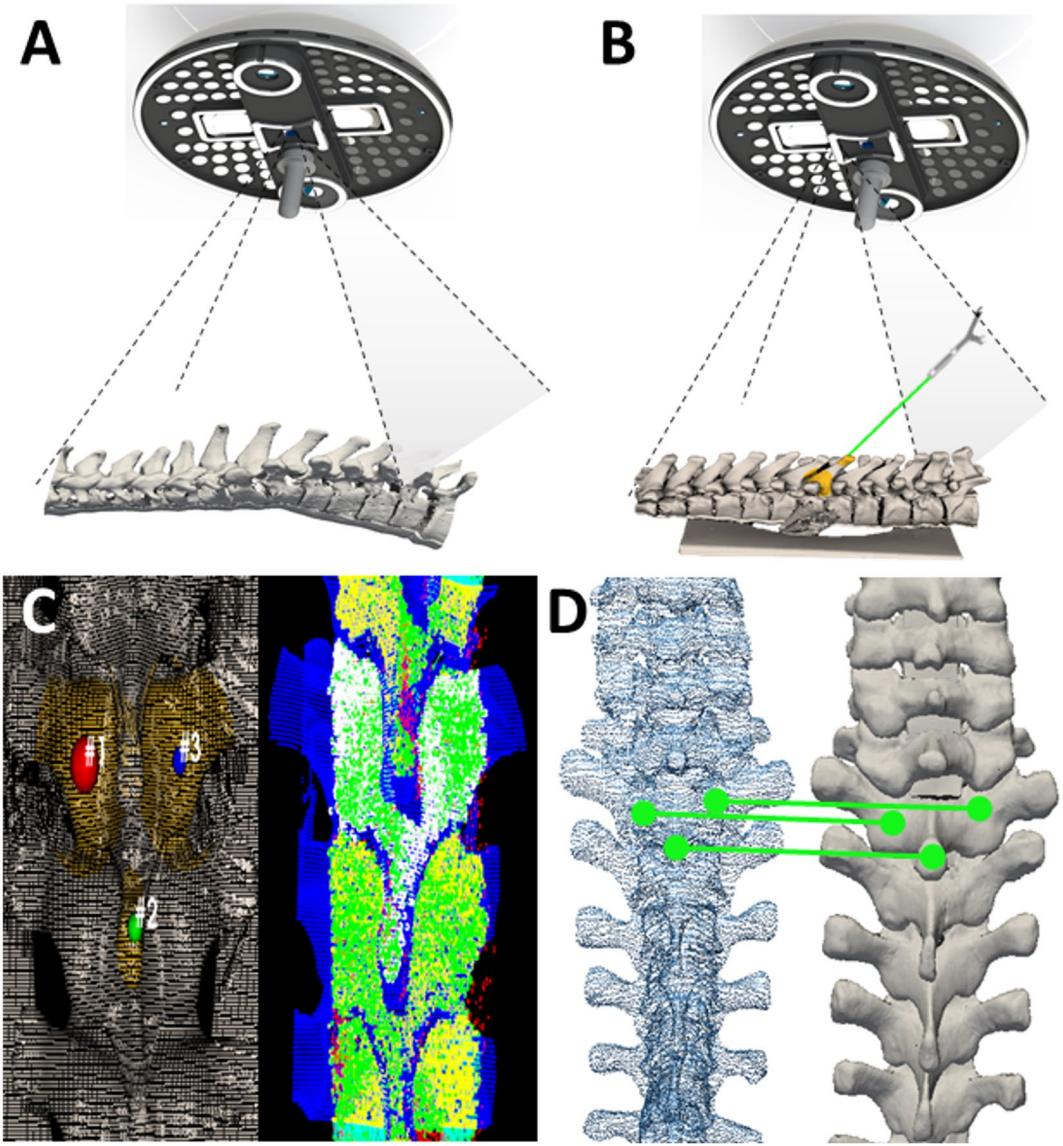
Optical topographical imaging (OTI) experimental navigation system. (**A**) Structured light patterns projected into the open surgical field. Structured light patterns deflect and deform upon reaching the surface of the target. Pattern deformations reflect height variations (along the optical axis) of the surface. (**B**) Registered reconstructed surface data to pre-acquired imaging data with tool tracking capabilities. Verification of the system’s accuracy is conducted by sliding a passively tracked probe along boney landmarks of the anatomy and confirming the system is reporting the tool’s spatial location correctly. (**C**) Grey-scale stereoscopic cameras acquire surface images: light patterns are projected onto the surface, images are captured and 3D reconstructions and thresholded point-clouds are created representing the bony surface of the spine. (**D**) Registration of the acquired 3D-point cloud to pre-acquired imaging data (i.e. CT, MRI, OTI) using an iterative closest point (ICP) algorithm based on a three-point picking protocol.

Intraoperative surgical navigation has become the standard-of-care in cranial neurosurgery for the localization of subsurface structures, including neoplasms and vascular lesions, and for targeting of electrical implants to specific nuclei. While not as ubiquitous, navigation for spinal surgery has undergone significant evolution over the past decade. This technological advancement has been driven by need, with 410,000 spinal fusion procedures performed in the United States in 2008, a number expected to rise significantly over the next decades with an aging population^1,2^. While instrumentation is often used to facilitate osseous fusion, breach of screws outside the intended trajectory occurs in 12–40% of screws (Fig. 1)^3–6^. This may result, acutely, in neurovascular injury and, in the longer term, mechanical construct failure, causing potentially life or limb-threatening complications which may require costly revision surgery^7–9^. Computer-assisted navigation has been developed to improve the accuracy of screw placement at all spinal levels, reducing breach rates to under ten percent^4,10–14^. Navigation is also increasingly being applied to non-neurosurgical procedures, including hip and knee arthroplasties, oral and maxillofacial reconstructions, delicate otologic drilling, and open abdominal surgery^15–17^. We demonstrate a new surgical navigation technology, developed in our Biophotonics and Bioengineering Laboratory (BBL), using optical topographical imaging (OTI) to create virtual 3D surfaces of open surgical cavities, allowing surgeons to visualize internal structures relative to exposed surface anatomy (Figs 2,3). Our system completes full bony surface registration using graphics processing units (GPU) considerably faster than current systems, with comparable spatial resolution, sparing the patient from additional radiation exposure, reducing operating room time and costs, and minimizing disruption to surgical workflow.

Despite the apparent benefit of spinal surgical navigation in reducing breach rates, adoption of navigation as standard of care has been slow due to lengthy setup/registration times, steep learning curves, and interruption of surgical workflow^18–20^. Contemporary benchmark navigation systems employ a ‘matched-point’ registration protocol relying on surgeons to drag a pointed probe across exposed bony anatomy to map to a preoperative computed tomography (CT) scan. These protocols have steep learning curves and take three to five-fold longer per screw than traditional fluoroscopy, necessitating additional anesthetic and operating room time^21–23^. Matched-point registration protocols are also unable to account for variances in spinal anatomy due to changes in patient positioning from CT gantry to operating table, critical in trauma and deformity cases. While this may be overcome with intraoperative 3D fluoroscopy or CT, it is at the cost of significant capital expense, increased patient radiation burden particularly with multilevel fusions, and substantial setup time^24–26^. Our experimental navigation system confers significant benefit over the currently available navigation techniques, implementing a simple point picking protocol and ultrafast optical radiation-free imaging registration to fuse the intraoperative surface anatomy with the preoperative CT. Rapid repeat registration allows for sequential segmental registration, minimizing intersegmental deviation from preoperative imaging to intraoperative positioning that would otherwise have required an intraoperative imaging device. While we validate our system here for craniospinal navigation, the technology is immediately applicable to non-neurosurgical navigation applications, with rapid repeat registration lending itself well to future soft-tissue applications.

## Results

*Ex-vivo* feasibility of our experimental optical navigation technology was studied in 6 adult human cadavers, resulting in the integrated design of navigation with surgical lighting. *In- vivo* proof-of-principle validation of OTI was performed on 10 anaesthetized ventilated adult swine models, where interference between optical illumination for surgical lighting and OTI, both in the visible spectrum, were studied and minimized. Optical imaging of subperiosteal dissection planes between soft and bony tissues, cluttered by bleeding and carbonization effects from electro-cautery using standard surgical techniques, was performed to demonstrate pre-clinical applicability and establish required specifications for 3D imaging speed (<0.5 seconds to acquire the entire operative field using standard surgical suction to clear pooling blood) and for maximal tolerated animal anatomy movement speed (<2 mm/second) using IR tracking. To study navigation accuracy, 71 thoracic and lumbar pedicle screws were inserted and quantified by comparing intraoperative trajectory data to true screw placement based on postoperative CT imaging. Median (95%) translational and angular error of the experimental navigation system in the adult swine model was 1.67 mm (5.12 mm) and 4.37° (12.95°) in the axial plane and 1.63 mm (7.81 mm) and 6.50° (17.76°) in the sagittal plane. Following engineering optimization based on the cadaver and swine data, human clinical trials were commenced for cranial and spinal surgical procedures. At the time of writing, 171 human craniospinal surgical procedures have been performed using our experimental navigation system. Relative displacement drift between stereo-cameras over time was found to degrade navigation accuracy during the validation phase, aggravated by larger thermal expansion coefficients of the 3D printed plastic material used in the experimental system. An active calibration protocol was developed to account for camera drift which showed statistically improved cranial screw coordinate measurements.

**Figure 4:**
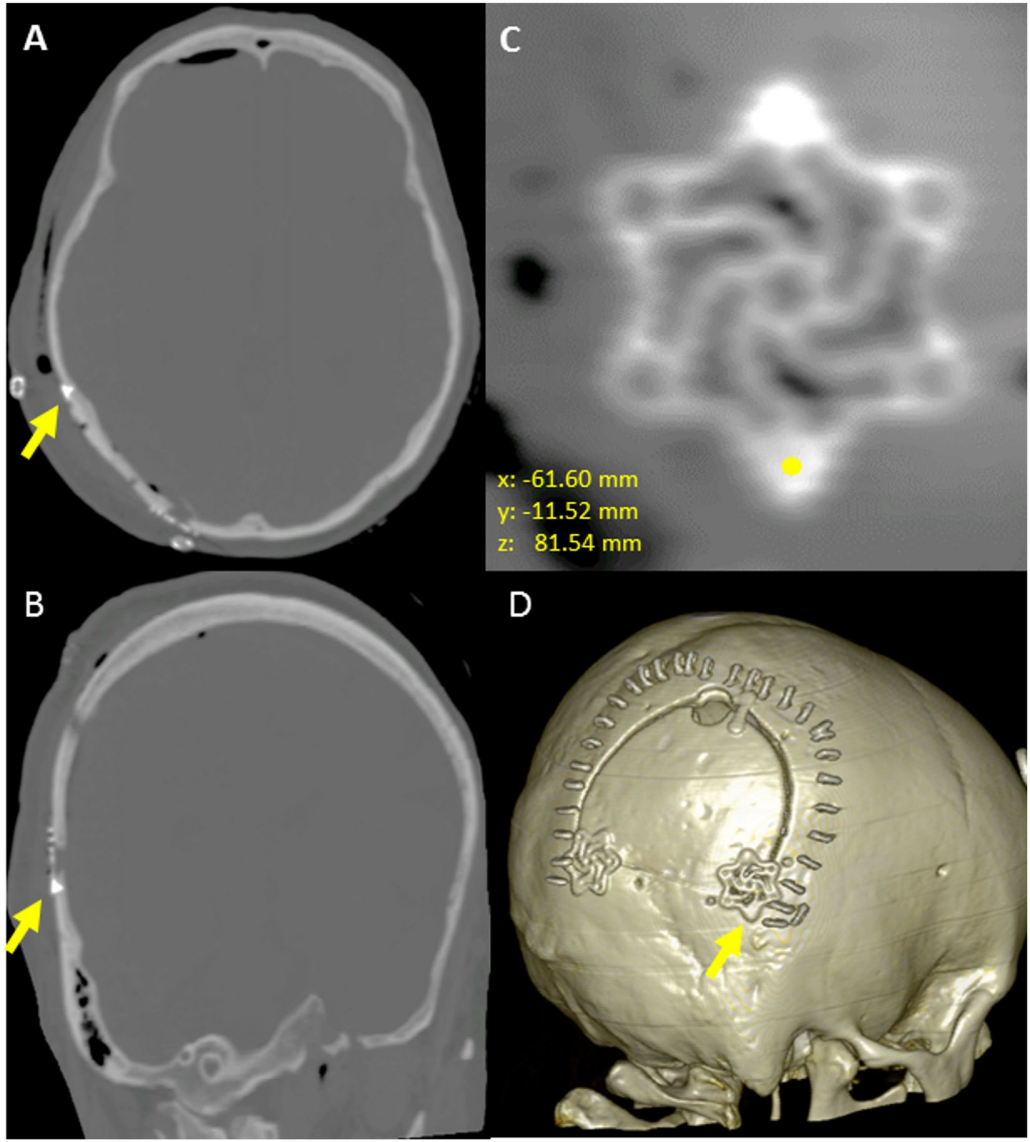
Engineering analysis quantifying cranial translational error. Comparison of postoperative cranial screw co-ordinates to intraoperative co-ordinates base on the location of the tracked probe. (**A**) Axial CT representation, (**B**) Coronal CT representation (**C**) Multiplanar reformatted CT image (2 cranial fixation screws), (**D**) 3-dimensional volume rendered CT.

In regards to cranial accuracy, Euclidean (3D) translational error was quantified based on the location of cranial screws fastening cranial fixation plates as measured on postoperative CT, relative to the intraoperative screw head location as reported by the experimental navigation system (Fig. 4). Median (95%) 3D translational errors for all cranial screws (N = 216 screws, 19 patients) was 2.49 mm (5.53 mm) with 1.18 mm (3.16 mm), 1.11 mm (3.95 mm), 1.15 mm (4.32 mm) in the X, Y, and Z directions respectively. Significant improvements in all axes were seen following software integration of the active calibration algorithm to account for camera drift over time (p < 0.001), reducing the median (95%) 3D translational error from 4.01 mm (5.99 mm) (N = 102 screws, 8 patients) without active calibration to 1.89 mm (3.40 mm) (N = 114 screws, 11 patients) with active calibration. Similarly, integration of the active calibration algorithm reduced the X, Y, and Z translations from 1.54 mm (4.08 mm), 1.97 mm (4.79 mm) and 1.65 mm (4.81 mm) to 0.89 mm (2.37 mm), 0.89 mm (2.19 mm), and 0.82 mm (2.13 mm). The relatively short length (4–5 mm) of the cranial screws precluded reliable angular deviation measurement.

**Figure 5:**
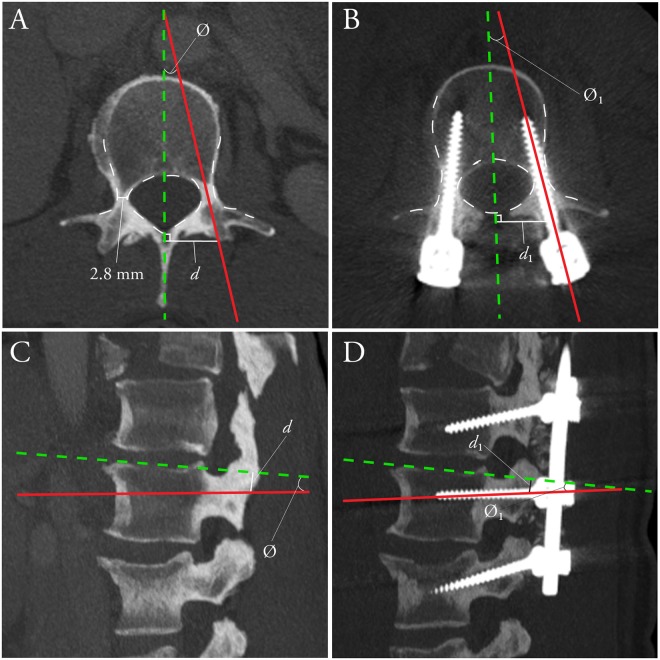
Engineering analysis quantifying absolute translational and angular deviation in the axial and sagittal planes. Example shown of a patient with hypoplastic pedicles at L2. (**A**) Intraoperative predicted screw trajectory (red) as visualized on a preoperative axial CT. (**B**) Postoperative actual screw trajectory (red) as visualized on a multiplanar reformatted postoperative CT. Axial distances (d) were measured at 90° relative to midsagittal axis (green line). Angle (Ø) represents corresponding trajectory angles. (**C**) Intraoperative predicted screw trajectory (red) as visualized on a preoperative sagittal CT. (**D**) Postoperative actual screw trajectory (red) as visualized on a multiplanar reformatted postoperative CT. Sagittal distances (d) were measured at 90° relative to the inferior or superior endplate (green line). Angle (Ø) represents corresponding trajectory angles. Errors in each plane were calculated as d_1_-d (translational) and Ø_1_-Ø (angular).

In regards to spinal surgical accuracy, previous work for spinal image-guided navigation techniques has focused on anatomic screw location (screw grading) rather than true *in-vivo* quantification of error (translational or angular deviations of the true screw trajectory vs. the intraoperative trajectory). In this study, translational and angular deviations were quantified for both the benchmark navigation systems and the experimental navigation system in the axial and sagittal planes (Fig. 5, Table 1). Absolute error measurements were quantified based on postoperative CT scans of implanted pedicle screws, ranging from 4.5–6.5 mm in diameter. Metallic CT artefacts amplify variation in absolute error measurements, particularly from large-diameter pedicle screws appropriate for our thoracolumbar series. The true engineering accuracy of the experimental navigation system is therefore underestimated. Clinical grading of screws, using the established Heary grading system^27^, was performed independently by two neuroradiologists (CH, PH), three neurosurgeons (DWC, NP, & LC) and two orthopedic spine surgeons (RG, GT) (Table 2). A mean clinical major breach (Heary III–IV) rate of 5.7% for all navigated screws was reported, with strong intraclass correlation (ICC: 0.725; p < 0.001) and fair inter-rater agreement (Fleiss’ Kappa (95% CI): 0.248 (0.243–0.254); p < 0.0001). Furthermore, there were no significant differences in major screw breaches between the experimental and benchmark systems (6.8% vs. 5.3%, p = 0.99).

**Table 1:** Generalized linear model: error vs. screw location and navigation method.

Clinical Variables	Screw Location and Error Median (95 percentile)					Navigation Method Median (95 percentile)		
	Cervical N = 9	Thoracic N = 225	Lumbar N = 130	Sacral N = 6	P-Value	Benchmark N = 209	Experimental N = 162	P-Value
Axial Distance Error (mm)	1.00 (1.73)	1.05 (3.69)	1.53 (3.94)	1.03 (1.93)	0.381	1.14 (3.92)	1.21 (3.42)	0.597
Axial Angle Error (deg)	2.79 (4.33)	2.07 (8.07)	2.48 (9.47)	6.05 (11.59)*	0.009*	2.43 (8.97)	2.15 (8.14)	0.839
Sagittal Distance Error (mm)	1.32 (2.20)	0.86 (1.28)	1.28 (4.12)	0.84 (2.13)	0.437	0.83 (3.62)	1.13 (4.25)	0.214
Sagittal Angle Error (deg)	1.55 (4.22)	2.57 (9.75)	2.47 (9.48)	3.86 (10.77)	0.485	2.60 (10.06)	2.33 (8.59)	0.492

Statistical analysis using generalized linear model of axial distance, axial angle error, sagittal distance error, and sagittal angle error as a function of screw location (cervical, thoracic, lumbar, and sacral) and navigation method (benchmark navigation, experimental navigation). No significant differences were seen using the experimental navigation system. Screws located in the sacrum demonstrated increased axial angle error vs. thoracic and lumbar spine.

**Table 2:** Clinical Heary grading of all navigated screws.

Grade	Reviewer 1	Reviewer 2	Reviewer 3	Reviewer 4	Reviewer 5	Reviewer 6	Reviewer 7
1	284 (98.3%)	266 (92.0%)	243 (84.1%)	220 (76.1%)	269 (93.1%)	259 (89.6%)	223 (77.2%)
2	4 (1.4%)	5 (1.7%)	32 (11.1%)	39 (13.5%)	7 (2.4%)	20 (6.9%)	36 (12.5%)
3	0 (0.0%)	7 (2.4%)	6 (2.1%)	15 (5.2%)	7 (2.4%)	2 (0.7%)	11 (3.8%)
4	1 (0.3%)	11 (3.8%)	8 (2.8%)	15 (5.2%)	6 (2.1%)	8 (2.8%)	19 (6.6%)

Clinical Heary grading of all navigated screws by 7 independent reviewers (two neuroradiologists, three neurosurgeons, and two orthopaedic spine surgeons). Grade I: the screw is entirely contained within pedicle; Grade II: the screw violates lateral pedicle but screw tip is contained within the vertebral body; Grade III: the screw tip penetrates anterior or lateral vertebral body; Grade IV indicates a medial or inferior breach of the pedicle; Grade V: screw tip or shaft violates pedicle or vertebral body, and endangers spinal cord, nerve root, or great vessels, requiring immediate revision.

**Figure 6:**
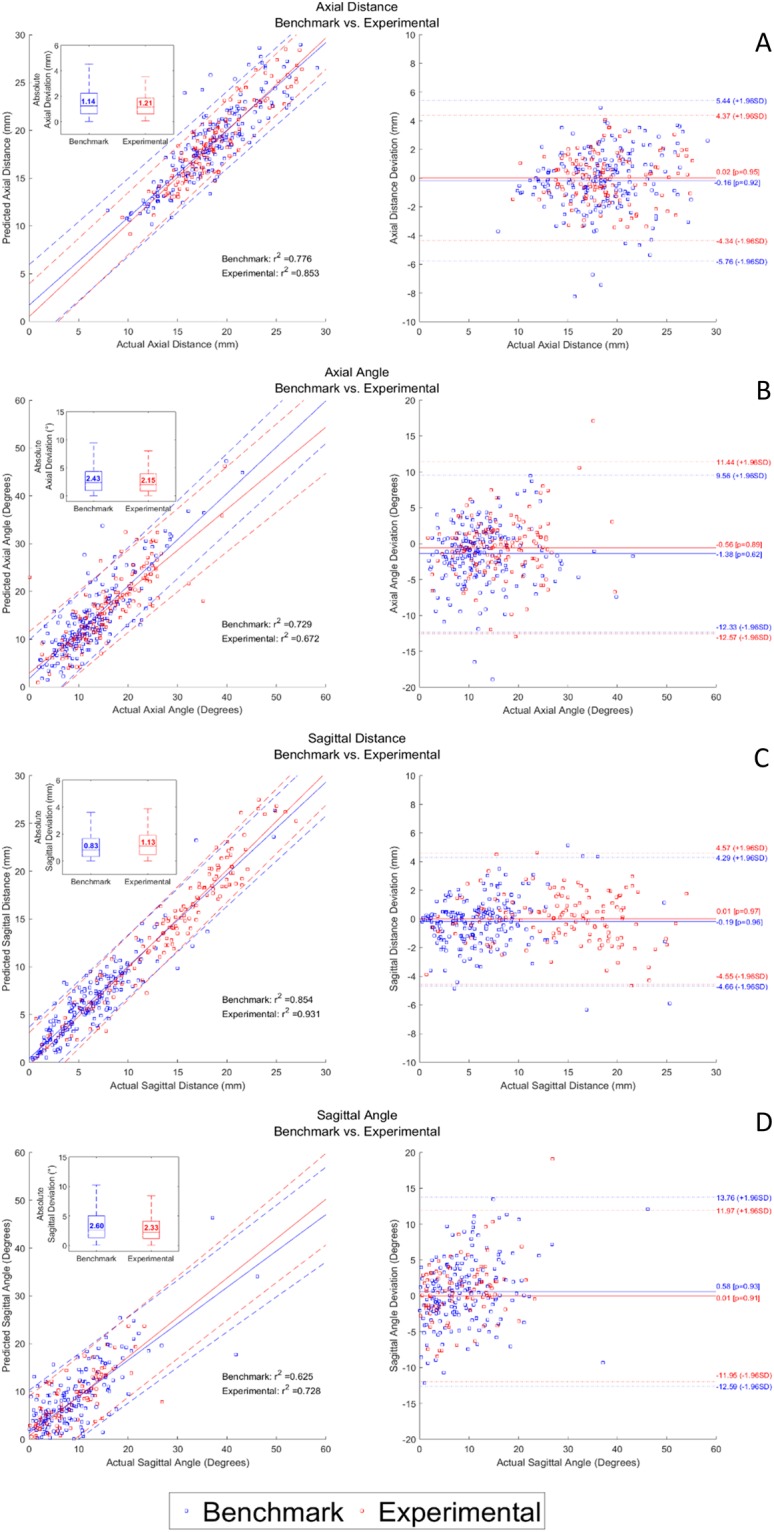
Bland Altman analysis. Left Panel: Correlation plots with corresponding boxplots comparing predicted intraoperative screw trajectory with actual postoperative screw trajectory for benchmark (blue) and experimental (red) navigation systems. Right Panel: Bland-Altman plots comparing actual screw trajectory with distance and angular deviations for (**A**) Axial Distance, (**B**) Sagittal Distance, (**C**) Axial Angle, (**D**) Sagittal Angle. No statistically significant differences were found.

In human clinical trials for spinal procedures, median (95%) translational and angular errors for benchmark systems (209 screws) were 1.14 mm (3.92 mm) and 2.43° (8.97°) in the axial plane and 0.83 mm (3.62 mm) and 2.60° (10.06°) in the sagittal plane, vs. 1.21 mm (3.42 mm) and 2.15° (8.14°) and 1.13 mm (4.25 mm) and 2.33° (8.59°) for the experimental navigation system (N = 162 screws) (Fig. 6). More navigated screws were inserted with benchmark system guidance prior to use of the experimental system, to ensure the surgical team’s proficiency in using existing commercial systems and reduce bias in the comparison. Without active calibration for spinal screw navigation (N = 50 screws), the median (95%) translational and angular errors were 1.27 mm (3.03 mm) and 1.88° (7.62°) in the axial plane and 1.60 mm (4.39 mm) and 2.33° (8.16°) in the sagittal plane, vs. 0.99 mm (3.37 mm) and 2.28° (8.09°) and 0.94 mm (2.56 mm) and 2.48° (7.79°) for navigation with active calibration (N = 79 screws). These differences did not reach statistical significance.

Univariate analysis, accounting for age, gender, surgical navigation method (i.e. benchmark navigation, experimental navigation) and screw location (cervical, thoracic, lumbar, sacrum) identified age as a predictor for increased axial distance error, and sacral screw location as a predictor of increased axial angle error vs. the thoracic and lumbar spine. Generalized linear regression confirmed sacral screw location as a predictor of increased axial angle error (p = 0.009; Table 1) and advanced age as an indicator of increased axial distance error (p = 0.005). Surgical workflow improvement, as measured by setup and registration time to enable navigation, was compared between the experimental and benchmark systems. For cranial procedures, the median total intra-operative setup and registration time interquartile range (IQR) for the experimental navigation system was 38 s (26–76) vs. 96 s (65–120) for the benchmark system (p = 0.001). For spinal procedures, experimental navigation system median (IQR) time was 41 s (25–68) vs. 258 s (143–355) for matched-point based (p < 0.001) and 794 s (609–1136) intraoperative CT based benchmark systems (p = 0.001) (Fig. 2D). Time for pre-operative data loading/processing was not measured, however is on the order of <5 minutes, with perhaps a few minutes of time savings for the experimental vs. matched-point based systems due to eliminating the need to select 6–10 points pre-operatively. This suggests that intraoperative navigation confers significant accuracy benefit compared to freehand and fluoroscopy techniques, and that the remarkable gains in surgical workflow facilitated by the experimental navigation system do not come at the expense of surgical accuracy.

## Discussion

This work represents a major shift in the current surgical paradigm through the introduction of ultra-fast optical topographical imaging and registration. We have demonstrated the implementation of an optical topographical imaging modality in craniospinal surgery, with thorough clinical and engineering data analysis to ensure surgical accuracy. By using optical imaging based surface point acquisition and GPU based parallel computing processing, we perform registration of intraoperative anatomy to preoperative MRI or CT imaging at speed orders of magnitude faster than current point-matching navigation systems. The form factor of our experimental surgical navigation system has been designed to integrate into the existing operating room environment, with the benefit of performing imaging and registration tasks considerably faster than existing technologies. While the navigation optics are integrated into an overhead surgical lighting unit, this is readily moved out of the surgical field following registration to maintain instrument tracking, hence functioning well even with a microscope in the field. We believe these significant innovations eliminate the workflow restrictions that have traditionally led some surgeons to forgo navigation in favor of freehand approaches.

While the utility of the present study is apparent in the context of craniospinal procedures, the same optical topographical imaging technology is suitable for a variety of applications. In the immediate-term, this technology may be readily translated to open lateral skull base approaches. The accuracy of surface mapping in this context via manual tracer digitization has been described^28^. Optical topographical imaging would allow significantly faster automated surface registration for open lateral skull base procedures, while maintaining functionality using an operating microscope. In the short-term, rapid optical topographical imaging allows for frequent repeat registrations, minimizing the significant target registration errors seen with existing neuronavigation technologies as a result of progressive brain shift during the procedure^29^. Frameless stereotactic navigation is also employed routinely in otolaryngology, with growing applications in orthopedic, abdominal, and craniomaxillofacial procedures^15,30–32^. The utility of optical topographical imaging techniques is evident in these non-neurosurgical applications, particularly in those with significant soft-tissue manipulation or deformation, where rapid repeat registration is required to maintain accurate correlation to preoperative imaging. High frequency re-acquisition of intraoperative optical images also lends itself well to augmented-reality, with co-registered images overlaid onto operating microscopes or other displays employed commonly in multiple surgical disciplines. More speculatively in the longer-term, applications of optical topographical imaging to endoscopic procedures may be considered. Miniaturization of a structured-light projection and imaging device into an endoscope form factor has been described previously^33^. However, significant work is required to enable accurate registration in the context of blood and mucous membranes present in a typical endoscopic endonasal skull base approach, and to maintain registration accuracy throughout the procedure.

The salient findings of our study are, first, that intraoperative navigation based on OTI is accomplished significantly faster than existing technologies. These differences are particularly pronounced when compared to newer-generation devices employing intraoperative CT scanning, relative to techniques requiring point-matching registration to preoperative imaging (Fig. 2D). Such benefit was enabled by the efficient GPU algorithm, as demonstrated by the computation time for optical image acquisition and registration at 5.07 ± 1.83 seconds measured over 476 craniospinal registrations, each consisting of over 250,000 surface points, with average throughput of over 52,000 points per second, representing an improvement on the current clinical paradigm, whereby spatial accuracy is maintained while vastly improving registration time and workflow. Second, for spinal procedures, absolute translational and angular accuracy of intraoperative navigation is comparable to benchmark technologies (Fig. 6). The accuracy measured in this study is the total surgical application accuracy, stacking the navigation system’s accuracy with the surgeon’s ability to utilize 3D navigation data in placing pedicle screws, where surgeon’s experience and anatomical knowledge also contribute^27^.

For cranial applications, the speed advantage of OTI relative to benchmark systems remains albeit less pronounced than in the spine. Multiple techniques for frameless stereotactic patient-to-image cranial registration have been developed, including surface-based or point-matching using anatomic landmarks, skin adhesive markers or bone-implanted fiducials. The accuracy of the OTI system in our cranial cohort is on par with that achieved using bone-implanted skull fiducials by current frameless navigation systems, and superior to the accuracy obtained using more common registration techniques such as surface or point matching on the scalp^34^. Remarkably, the accuracy of OTI is also on par to that reported for frame-based stereotactic localization, the current gold-standard for cranial navigation, which ranges from 2.5–3.5 mm in 3D error in both phantom and *in-vivo* studies^35–37^. With the introduction of software improvements of the active calibration protocol adjusting for camera drift over time, a significant improvement in median (95%) 3D accuracy from 4.01 mm (5.99 mm) to 1.89 mm (3.40 mm) was seen, without using bone-implanted fiducial screw based registration^34^ which would disrupt existing surgical workflows.

The introduction of the active calibration protocol is crucial as it facilitates rapid intraoperative re-registration without exposing the patient to additional radiation. While the exact cause of camera drift is unknown, several possible factors have been identified including thermal expansion of the aluminum and acrylonitrile butadiene styrene (ABS) camera housing due to heat produced by the LED surgical lights, optical drift occurring within the cameras, and torsion stemming from the structural design of the experimental system and various screw connection sites. These inaccuracies, while still present in spine, are more prominent in cranial applications where a significant time lag between registration and screw placement exists, during which surgical steps such as mechanical drilling, cutting, musculocutaneous flap traction, and patient movement can all introduce navigation error due to relative displacement between the patient’s cranium and the navigation reference clamp. The active calibration protocol obviates the need for additional intraoperative imaging while maintaining the required surgical accuracy. While the current analysis comprises the experience of a number of surgeons, the majority of screws navigated with the experimental system were either directly placed or supervised by one surgeon (V.X.D.Y.), representing a single-surgeon influence. Larger studies, involving multiple surgeons, are therefore underway to fully evaluate the evolution of a novice user to a skilled operator using the experimental navigation system, with multicenter studies representing the subsequent logical progression.

## Methods

### System Design

Our experimental navigation system consists of a small camera gantry integrated with surgical lighting. The camera gantry is composed of two cameras, a digital micromirror device pico-projector and an infrared optical tracking system (Fig. 2A,B). The projector illuminates a patterned light of known structure and periodicity (e.g. grids, repeating bars) that is recorded by the cameras and used to reconstruct the 3D surface of the patient (Fig. 3A)^38^. The patterns enable image correspondence between the stereo cameras to be established. With calibrated cameras, the stereo images allow for 3D mapping of the surface correlating to the various height disparities. The experimental navigation system registers the acquired 3D-point cloud to pre-acquired imaging data (i.e. CT, MRI) using a surface registration algorithm that is based on the iterative closest point (ICP) algorithm (Fig. 3C,D). The experimental navigation system also utilizes an infrared (IR) tracking system containing two IR cameras surrounded by IR LEDs to illuminate the tracking volume. The IR system is currently used to track surgical tools using passive-reflective markers (Fig. 3D).

### Pre- and Intra-Operative Workflow

Pre-operatively, the pre-acquired imaging data (CT/MRI) must be loaded onto the experimental navigation system, as with current benchmark systems registered to pre-acquired imaging. A DICOM (Digital Imaging and Communications in Medicine) stack is loaded via disc/USB/Ethernet, and thresholded appropriately by the user to maximize visible bony landmarks while minimizing noise. For spinal procedures, the spinal level to be registered is pre-identified. Intra-operatively, the navigation system is introduced into the surgical field as a standard overhead surgical light (Video 1). Following clamping of the dynamic reference frame to the target spinal level, registration proceeds by selecting three approximate points on the pre-identified level for registration, followed by structured-light acquisition of the 3D surface of the exposed field (Video 1). Registration proceeds similarly for cranial procedures. In its current iteration, OTI registration is not continuously renewed during the surgical procedure. Following registration, if a surgical microscope is required for the procedure, the navigation system head can be moved out from its overhead position and aimed towards the reference frame, as with current benchmark optical navigation systems, to maintain registration and allow tool tracking with a microscope in the field.

### Human, Cadaver, and Animal Research

This work comprises cadaver validation (Sunnybrook Health Sciences Centre Research Ethics Board #260-2011), animal studies (Sunnybrook Health Sciences Centre Animal Use Protocol #13-512), and human trials including prospective and retrospective analysis of pedicle screw placement and cranial surgery using neuronavigation (Sunnybrook Health Sciences Centre Research Ethics Board #177-2013, #309-2014, #086-2015, #004-2015). All animal research was performed in accordance with relevant institutional guidelines and regulations. Informed consent was obtained for all human subjects. Clinical trials were registered at clinicaltrials.gov (Phase I: NCT03391024 – 24/9/2013, Phase II: NCT03391011 – 13/3/2015, & Phase III: NCT03391089 – 21/1/2016).

### Human trials

Reporting of all human clinical trials is in accordance with the guidelines for **ST**rengthening the **R**eporting of **OB**servational studies in **E**pidemiology (STROBE – www.strobe-statement.org).

### Clinical Trial Design

To maximize safety, the clinical trials begin with a lead-in phase where the experimental system was used to obtain optical topographical imaging and perform registration only (recruiting 09/2013–08/2014), followed by a validation phase where the benchmark navigation system provided surgical guidance while the experimental system measured established guidance trajectories (recruiting 03/2015–10/2015), a cross-over phase where the roles of the benchmark and experimental systems were switched (recruiting 1/2016–10/2016), and finally using the experimental navigation system only for surgical guidance with post-operative imaging analysis to verify accuracy (recruiting 11/2016 – present). Following engineering optimization based on our swine data, human clinical trials were commenced for craniospinal procedures. Human trials were benchmarked against two commercial point matching-based navigation systems – NAV3/3i (Stryker; Portage, MI, USA), and StealthStation S7 (Medtronic Sofamor Danek; Memphis, TN, USA), for cranial procedures using the point merge and/or surface tracer protocols. For spinal procedures, benchmark systems consisted of NAV3/3i using point merge and surface tracer protocols, or StealthStation S7 in conjunction with O-arm for automated registration to intraoperative imaging. 3D Navigation was used to guide thoracolumbar spinal pedicle screw insertion, and map the skull projections of intracranial lesions to optimize craniotomy boundaries. At the time of writing, 171 craniospinal surgical procedures have been performed using our experimental optical navigation system comprising 476 registrations. Inclusion criteria for all human clinical trials were as follows: >18 years of age, with no history of prior surgery in the area to be operated upon (cranial or spinal, as appropriate).

### Engineering and Clinical Analysis

#### Experimental Navigation System - Animal Cohort

Translational and angular deviation in the axial and sagittal planes were analyzed for 71 thoracic and lumbar pedicle screws. Screws were placed using the experimental navigation system.

### Experimental Navigation System - Cranial Cohort

Accuracy was quantified by placing a tracked probe on the screws of fixed cranial plates and comparing the reported position of the tooltip to the coordinates of the cranial screws on a post-operative CT registered to the pre-operative image (Fig. 4). Data were obtained for 216 cranial screws. Subsequent to the integration of an active calibration algorithm utilizing a known geometry to apply appropriate transformations, cranial screws were dichotomized into active calibration (N = 102) vs. non-active calibration (N = 114).

### Image Post-Processing

Post-operative CT images were co-registered to pre-operative CT using an iterative closest point (ICP) algorithm. The location of the cranial screw was determined as the point where the center of the screw comes into contact with the skull. The screw location recorded by the OTI system was compared to the location of the centre of the screw on post-operative CT. The post-operative actual screw location was located using an OSIRIX 64 bit workstation (Version 10.9.5, PIXMEO SARL, Switzerland).

### Engineering Analysis

Application accuracy was compared between screws that were inserted with and without active calibration using a Mann-Whitney univariate test. p < 0.05 was considered statistically significant.

### Benchmark Navigation System – Clinical Spine Cohort

Translational and angular deviation in the axial and sagittal planes were computed for 209 cervical, thoracic and lumbar pedicle screws. Screws were placed using commercially available navigation systems. All screws were graded clinically using the Heary Grading System.

### Experimental Navigation System – Clinical Spine Cohort

After the lead-in phase (which included freehand or fluoroscopy guided screws, N = 21 screws), translational and angular deviation in the axial and sagittal planes were measured for 162 screws, which were placed using commercially available benchmark navigation or OTI navigation.

### Image Post-Processing

Pre- and post-operative CT images were resliced to 0.3 mm thickness and dynamically resliced using multiplanar reconstruction corresponding to the axial and sagittal co-ordinates of the intra-operative and post-operative screw trajectories. For benchmark spine screws, the corresponding axial and sagittal co-ordinates were localized manually using distinct anatomical landmarks. The entry point of the screw was determined as the point where the center of the screw comes into contact with the vertebral body. The distance from the axis of symmetry perpendicular to the point of entry, as well as the angle between the screw trajectory and the perpendicular distance of the entry point, were recorded on the pre- and post-operative axial and sagittal multiplanar reconstructions. All measurements were performed using an OSIRIX 64-bit workstation (Version 10.9.5, PIXMEO SARL, Switzerland) (Fig. 5).

### Clinical Grading

All screws were graded clinically using the method introduced by Heary *et al*.^39^. where Grade I denotes the screw is entirely contained within pedicle; Grade II the screw violates lateral pedicle but screw tip is contained within the vertebral body; Grade III indicates the screw tip penetrates anterior or lateral vertebral body; Grade IV indicates a medial or inferior breach of the pedicle; Grade V involves a violation of the pedicle or vertebral body endangering the spinal cord, nerve root, or great vessels. Clinical grading was performed independently by two neuroradiologists (CH, PH), three neurosurgeons (DWC, NP, & LC) and two orthopedic spine surgeons (RG, GT) (Table 2). Heary grades were assessed for inter-rater reliability using the intra-class correlation coefficient. Clinical grades were dichotomized into no breach/minor breach (Heary Grade ≤2) vs. major breach (Heary Grade >2) and reliability of agreement was measured using the Fleiss’ Kappa test.

### Engineering Analysis

Engineering analysis was performed as previously outlined by Jakubovic *et al*.^40^. Translational and angular deviations in the axial and sagittal planes in the benchmark spine cohort were compared with the corresponding deviations in the experimental navigation system spine cohort. Statistical analysis was not performed on the lead-in phase as the experimental navigation system was not used for guidance. Translational and angular deviations were compared using the Kruskal-Wallis test, visualized on Bland-Altman plots and tested using a generalized linear model. Age, gender, screw location, and guidance method (navigation, fluoroscopy, or freehand), were considered as covariates. p < 0.05 was considered statistically significant. All statistical analyses were performed using IBM SPSS 17.0 statistical software.

## Electronic supplementary material

Video 1

Supplementary Video 1 Legend

## Data Availability

The datasets generated during and/or analysed during the current study are available from the corresponding author on reasonable request.
