# Supplementary material for: High Speed, High Density Intraoperative 3D Optical Topographical Imaging with Efficient Registration to MRI and CT for Craniospinal Surgical Navigation
Source: Sci Rep. 2018 Oct 5;8:14894. doi: 10.1038/s41598-018-32424-z (PMC6173775; doi:10.1038/s41598-018-32424-z)
Supplement: Supplementary file 2 — Supplementary Video 1 Legend [file 41598_2018_32424_MOESM2_ESM.docx]

High Speed, High Density Intraoperative 3D Optical Topographical Imaging with Efficient Registration to MRI and CT for Craniospinal Surgical Navigation

Raphael Jakubovic PhD^1,2†^, Daipayan Guha MD^2,3,4†^, Shaurya Gupta^2,3^, Michael Lu^2^, Jamil Jivraj MASc^2,5^, Beau A. Standish PhD^2^, Michael K. Leung Msc^2^, Adrian Mariampillai PhD^2^, Kenneth Lee PhD^2^, Peter Siegler PhD^2^, Patryk Skowron BSc^2,5^, Hamza Farooq BEng^2,5^, Nhu Nguyen BEng^2,5^, Joseph Alarcon BEng^2,5^, Ryan Deorajh BEng^2,5^, Joel Ramjist BSc^2,5^,Michael Ford MD, FRCSC^6^, Peter Howard MD,FRCSC^7,8^, Nicolas Phan MD, FRCSC^3^, Leo da Costa MD^3^, Chris Heyn MD, PhD, FRCPC^7,8^, Gamaliel Tan MBBS, FRCS^9^, Rajeesh George MBBS, MS^9^, David W. Cadotte MD, PhD, FRCSC^10,11^, Todd Mainprize MD, FRCSC^3^, Albert Yee MD, MSc, FRCSC, DABOS^6^, Victor XD Yang MD, PhD, PEng, FRCSC ^2,3,4,5^

^1^Department of Biomedical Physics, Ryerson University, Toronto, ON, Canada

^2^Biophotonics and Bioengineering Laboratory, Ryerson University Sunnybrook Health Sciences Centre, Toronto, ON, Canada

^3^Division of Neurosurgery, Department of Surgery, University of Toronto. Toronto, ON, Canada

^4^Institute of Medical Science, School of Graduate Studies, University of Toronto. Toronto, ON, Canada

^5^Department of Electrical and Computer Engineering, Ryerson University, Toronto, ON, Canada

^6^Division of Orthopedic Surgery, Department of Surgery, University of Toronto. Toronto, ON, Canada

^7^Division of Neuroradiology, Department of Medical Imaging, Sunnybrook Health Sciences Centre, Toronto, ON, Canada

^8^Department of Medical Imaging, University of Toronto. Toronto, ON, Canada

^9^Jurong Health, Ng Teng Fong General Hospital, Singapore, Singapore

^10^Spine Program and Division of Neurosurgery, Department of Clinical Neurosciences, Department of Radiology, University of Calgary, Calgary, Canada

^11^Hotchkiss Brain Institute, Cumming School of Medicine, University of Calgary, Calgary, Canada

**Corresponding author:**

Victor XD Yang, MD PhD PEng FRCSC

Canada Research Chair, Biophotonics and Bioengineering Laboratory

Division of Neurosurgery/Brain Sciences Program

Sunnybrook Health Sciences Centre/Sunnybrook Research Institute

University of Toronto/Ryerson University

2075 Bayview Avenue, Toronto, Ontario, M4N 3M5, Canada

Tel: 416-803-9320

Email: victor.yang@sunnybrook.ca

† Authors have contributed equally to this work and hold joint first authorship.

**Supplemental Files - Legend**

**Video 1**. High density structured light imaging from overhead surgical lighting provides efficient registration workflow in this study. Video illustrates 3D topographic image registration to pre-operative thoracic spinal CT scan, with sharp surgical tool tip in contact of bony surfaces for accuracy verification. An approximate 3-point picking for initial alignment (00:02-00:04) is followed by structured light 3D surface acquisition (00:05-00:06), automated registration to pre-operative imaging (00:08-00:09). An example of the acquired 3D point cloud is shown from 00:14-00:19.
